# STAG2 mutations regulate 3D genome organization, chromatin loops, and Polycomb signaling in glioblastoma multiforme

**DOI:** 10.1016/j.jbc.2024.107341

**Published:** 2024-05-03

**Authors:** Wanying Xu, Jung-Sik Kim, Tianyi Yang, Alvin Ya, Lisa Sadzewicz, Luke Tallon, Brent T. Harris, Jann Sarkaria, Fulai Jin, Todd Waldman

**Affiliations:** 1 Department of Genetics and Genome Sciences, Case Comprehensive Cancer Center, Case Western Reserve School of Medicine, Cleveland, Ohio, USA; 2 The Biomedical Sciences Training Program, School of Medicine, Case Western Reserve University, Cleveland, Ohio, USA; 3 Department of Oncology, Lombardi Comprehensive Cancer Center, Georgetown University School of Medicine, Washington, District of Columbia, USA; 4 MD/PhD Program, Georgetown University School of Medicine, Washington, District of Columbia, USA; 5 Tumor Biology Training Program, Georgetown University School of Medicine, Washington, District of Columbia, USA; 6 Institute for Genome Sciences, University of Maryland, Baltimore, Maryland, USA; 7 Departments of Neurology and Pathology, Georgetown University School of Medicine, Washington, District of Columbia, USA; 8 Department of Radiation Oncology, Mayo Clinic, Rochester, Minnesota, USA; 9 Department of Computer and Data Sciences, Department of Population and Quantitative Health Sciences, Case Comprehensive Cancer Center, Case Western Reserve University, Cleveland, Ohio, USA

**Keywords:** brain tumor, cancer biology, gene expression, tumor cell biology, tumor suppressor gene, chromatin, chromatin structure, STAG2, cohesin

## Abstract

Inactivating mutations of genes encoding the cohesin complex are common in a wide range of human cancers. STAG2 is the most commonly mutated subunit. Here we report the impact of stable correction of endogenous, naturally occurring STAG2 mutations on gene expression, 3D genome organization, chromatin loops, and Polycomb signaling in glioblastoma multiforme (GBM). In two GBM cell lines, correction of their STAG2 mutations significantly altered the expression of ∼10% of all expressed genes. Virtually all the most highly regulated genes were negatively regulated by STAG2 (*i.e.*, expressed higher in STAG2-mutant cells), and one of them—HEPH—was regulated by STAG2 in uncultured GBM tumors as well. While STAG2 correction had little effect on large-scale features of 3D genome organization (A/B compartments, TADs), STAG2 correction did alter thousands of individual chromatin loops, some of which controlled the expression of adjacent genes. Loops specific to STAG2-mutant cells, which were regulated by STAG1-containing cohesin complexes, were very large, supporting prior findings that STAG1-containing cohesin complexes have greater loop extrusion processivity than STAG2-containing cohesin complexes and suggesting that long loops may be a general feature of STAG2-mutant cancers. Finally, STAG2 mutation activated Polycomb activity leading to increased H3K27me3 marks, identifying Polycomb signaling as a potential target for therapeutic intervention in STAG2-mutant GBM tumors. Together, these findings illuminate the landscape of STAG2-regulated genes, A/B compartments, chromatin loops, and pathways in GBM, providing important clues into the largely still unknown mechanism of STAG2 tumor suppression.

Cohesin is a chromatin-bound ring complex that plays important roles in 3D genome organization, sister chromatid cohesion, and DNA repair (1). Although cohesin is known to control gene expression, the breadth, magnitude, and biological significance of this effect is controversial (2). Mutational inactivation of genes encoding components of cohesin is common in a wide variety of cancer types, including bladder cancer, myeloid leukemia, Ewing sarcoma, GBM, and others (3, 4, 5). STAG2 is by far the most commonly mutated subunit; truncating mutations of the STAG2 gene account for >50% of all cohesin mutations in cancer. The Cancer Genome Atlas has classified STAG2 as one of 12 genes significantly mutated in four or more cancer types (6).

Initial efforts to identify the mechanism of STAG2 tumor suppression focused on its canonical role in sister chromatid cohesion and resolution of sister chromatids during mitosis (3, 4). However, it was soon realized that some tumor-derived STAG2 mutants retained the ability to enforce normal sister chromatid cohesion and that many STAG2-mutant cancers maintained normal karyotypes (7, 8).

Several recent discoveries into the basic biology of cohesin have suggested exciting new potential mechanisms for STAG2 tumor suppression. In 2017 several groups demonstrated that various subunits of cohesin (but not STAG2) are required for the maintenance of the chromatin loops that underlie the complex packing structure of chromatin in the nucleus (referred to as 3D genome organization; refs. (9, 10, 11, 12)). Two years later the biochemical basis of this was reported – remarkably, that cohesin is actually the biochemical engine responsible for the generation (often referred to as “extrusion”) of those chromatin loops (13, 14).

These new fundamental discoveries have suggested that cancer-causing mutations in cohesin subunits such as STAG2 could cause cancer through an as-yet-undefined, likely subtle effects on the chromatin loops that underly 3D genome organization and that, at least in some cases, regulate the expression of nearby genes. While the specific effect(s) of tumor-derived STAG2 mutations on chromatin loop extrusion remains unknown, one clue has come from studies indicating that STAG1-containing cohesin complexes, which replace STAG2-containing cohesin complexes in STAG2-mutant cells, are more processive than STAG2-containing cohesin complexes during chromatin loop extrusion (15).

Another clue to the mechanism(s) of STAG2 tumor suppression has come from the study of the intersections of cohesin and Polycomb signaling in model organisms. Polycomb Group (PcG) proteins are chromatin remodeling proteins that enforce epigenetic silencing of gene expression by modulating histone methylation and 3D genome organization (16). Beginning in 2012, studies performed in *Drosophila* embryos suggested that PcG and cohesin complexes had interacting, yet opposing functions in the transcriptional regulation of developmentally relevant genes (17, 18). Then, in 2020, these findings were extended to mammalian (murine) cells (19, 20). These data from model organisms have suggested an additional possible new mechanism of STAG2 tumor suppression—that STAG2-containing cohesin complexes negatively regulate Polycomb signaling, an effect that is alleviated by tumor-derived mutations in STAG2.

In light of these recent clues into potential new mechanisms of STAG2 tumor suppression, we set out to define the effects of naturally occurring tumor-derived mutations of STAG2 on 3D genome organization, chromatin loops, and gene expression in GBM. We focused our studies on GBM because it is a devastating cancer with few therapeutic options, and because focusing on a single tumor type, instead of multiple tumor types with different epigenomic states, maximizes our ability to detect subtle effects of STAG2 mutations. These efforts have revealed that STAG2 controls 3D genome organization, chromatin loops, and Polycomb signaling in GBM.

## Results

### Identification of STAG2-regulated genes in GBM cells and tumors

**Figure 1:**
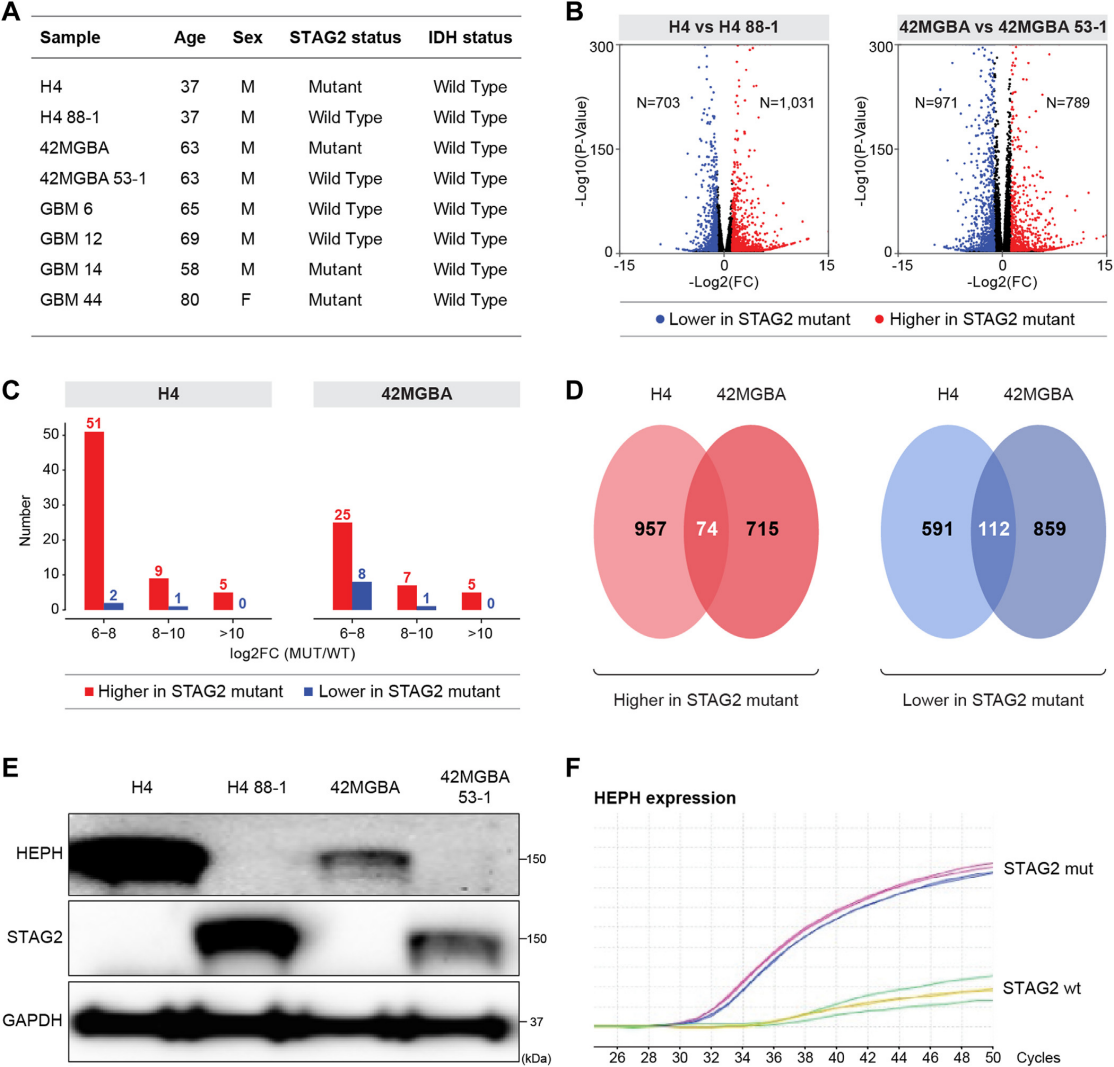
**Impact of STAG2 mutations on global gene expression in GBM cells.***A*, GBM cell lines and patient-derived xenograft (PDX) samples used in this study. *B*, volcano plots of RNA-seq data comparing (*left*) H4 parental cells (STAG2-mutant) with 88-1 STAG2-corrected derivatives and (*right*) 42MGBA parental cells (STAG2-mutant) and 53-1 STAG2-corrected derivatives. Differentially-expressed genes with *p* < 0.01 and |log2(fold-change)| >1 are in *red* (higher in STAG2-mutant) or *blue* (lower in STAG2-mutant). *C*, bar graphs indicate the numbers of the most highly differentially-expressed genes that are higher and lower in STAG2-mutant H4 (*left*) and 42MGBA (*right*) cells. All genes whose expression differed by equal to or more than 2^6^ (64-fold) between isogenic STAG2-mutant and wild-type cells are included. *D*, Venn diagrams indicating the numbers of STAG2-regulated genes conserved between the H4 and 42MGBA experimental systems. *E*, Western blot for HEPH and STAG2 in the H4 and 42MGBA cell systems, with GAPDH as the loading control. This experiment was performed twice. *F*, qRT-PCR analysis of HEPH expression in the STAG2 wild-type (*yellow, green*) and STAG2-mutant (*red, blue*) GBM PDX tumors. qRT-PCR for GAPDH indicated identical amounts of RNA in each sample (not shown).

To measure the effect of tumor-derived STAG2 mutations on global gene expression in GBM, we performed RNA-seq on two pairs of human GBM cell lines harboring different naturally occurring truncating mutations in STAG2 and their isogenic derivatives with their respective STAG2 mutations corrected by gene editing (Fig. 1*A*). H4 harbors the STAG2 357N>frameshift mutation (21) and 42MGBA harbors the STAG2 653S>Stop mutation (22) whereas H4 88-1 and 42MGBA 53-1 are their respective derivatives with the mutant allele of STAG2 corrected. We previously reported that the correction of mutant STAG2 in these cell lines restored normal levels of STAG2 expression and sister chromatid cohesion (3).

RNA-seq libraries were prepared from biological triplicates of each cell line to detect STAG2-regulated genes with high sensitivity (see Table S1 for reads/sample and Table S2 for complete RNA-seq data). Comparing H4 and H4 88-1 cells, 4% of genes (1031/23,272) were expressed higher in H4 cells whereas 3% (703/23,272) were expressed higher in H4 88-1 cells (Fig. 1*B*; *p* < 0.01 & |log2FC|>1). Comparing 42MGBA and 42MGBA 53-1 cells, 4% (789/22,405) of genes were expressed higher in 42MGBA cells whereas 4% (971/22,405) were expressed higher in 42MGBA 53-1 cells (Fig. 1*B*). Interestingly, when using more stringent fold-change cutoffs (>64-fold), the vast majority of the differentially expressed genes were expressed higher in the STAG2-mutant parental cells in both pairs (Fig. 1*C*). See Table S3 for a complete list of these most highly STAG2-regulated genes.

About 10% of all STAG2-regulated genes were conserved between the H4 and 42MGBA experimental systems (Fig. 1*D*; Tables S4 and S5), which is 2.2 and 4.0 times higher than would be expected by chance alone for genes expressed higher and lower, respectively, in STAG2-mutant cells (*p* < 0.01, hypergeometric test). These in-common STAG2-regulated genes were enriched for a number of different functional pathways, including several directly related to cellular proliferation (Fig. S1, *A–F*). qRT-PCR analysis of a subset of the most robust in-common STAG2-regulated genes confirmed the direction and general magnitude of the differential expression in every case (Table S6).

We next tested whether these qRT-PCR-validated STAG2-regulated genes were regulated by STAG2 in uncultured human GBM tumors as well. We prepared RNA from two STAG2-mutant and two STAG2 wild-type human GBM patient-derived xenografts (PDXs; Fig. 1*A*) and performed qRT-PCR for the 16 STAG2-regulated genes shown in Table S6. In most cases the expression levels did not correlate with the STAG2 mutational status of the PDXs, indicating that their regulation is complex, involving factors in addition to STAG2. However, the expression of the HEPH gene (hephaestin; putative iron transporter; ref. (23)) was STAG2-dependent in both the cell lines *and* the PDXs (Fig. 1, *E* and *F*). This finding, together with the recent observation that STAG2 can modulate sensitivity to iron overload (24), points to HEPH as a promising putative effector of STAG2 tumor suppression in GBM.

### Effects of STAG2 mutations on A/B compartment assignments in GBM

We next set out to define the effects of STAG2 mutations on 3D genome organization in GBM by performing Hi-C on biological replicates of the isogenic pairs of H4 and 42MGBA cells and the four human GBM PDXs (Table S7).

**Figure 2:**
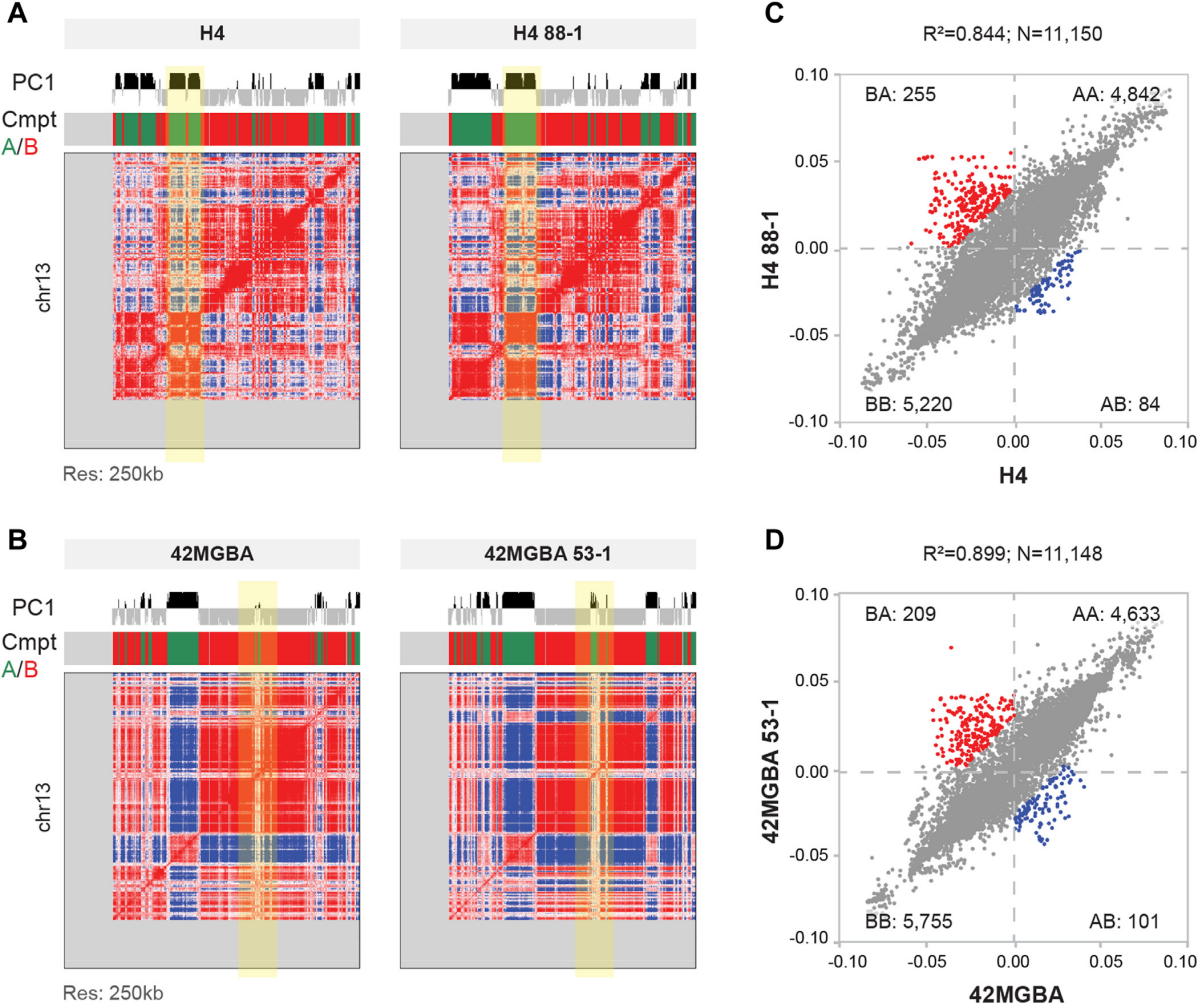
**Impact of STAG2 mutations on A/B compartment assignments in GBM cells.***A* and *B*, heatmaps showing the Hi-C correlation matrices at 250 kb resolution for (*A*) parental H4 cells and H4 88-1 STAG2-corrected derivatives and (*B*) parental 42MGBA cells and 42MGBA 53-1 STAG2-corrected derivatives. PC1 refers to Principal Component 1, which defines whether individual 250 kb bins are Compartment A or B. Chromosome 13 is shown. (A) compartments are *green* and (B) compartments are *red*. Loci highlighted in *yellow* have switched from (*B*) to (*A*) after STAG2 correction. *C* and *D*, scatterplot analysis depicting the effect of STAG2-correction on the compartment assignments of 250 kb bins in (*C*) H4 and (*D*) 42MGBA cells. Compartment switches with *p* < 0.01 are shown in *red* and *blue*. R-squared and the total number of bins tested (N) are shown at the top of each scatter plot.

We first analyzed the effect of STAG2 mutations on A/B compartment structure in the H4 and 42MGBA cells. A/B compartments are low-resolution features of 3D genome organization that define regions of open, transcriptionally active chromatin (A compartments) and closed, transcriptionally inactive chromatin (B compartments). There were substantial differences between these two cell lines, but the vast majority of compartment assignments were the same within each isogenic pair (a representative chromosome is shown in Fig. 2, *A* and *B*). In H4 cells, 84 (0.7%) A compartment bins switched to B, and 255 (2.3%) B compartment bins switched to A (Fig. 2*C*) after STAG2 correction. Similarly, in 42MGBA cells, 101 (0.9%) A compartment bins switched to B, and 209 (1.9%) B compartment bins switched to A (Fig. 2*D*) after STAG2 correction. Examples of A/B compartment switching after STAG2 correction in H4 and 42MGBA cells are highlighted in yellow in Figure 2, *A* and *B*, respectively.

Next, we determined whether any of the switched compartments were in common between H4 and 42MGBA cell systems. There were six 250 kb bins that switched from A to B and 25 bins that switched from B to A after STAG2 correction in both H4 and 42MGBA cells. Two of the conserved compartment switches, both from B to A, were comprised of multiple contiguous 250 kb bins - one immediately upstream of the STAG2 gene itself (Fig. S2, *A* and *C*) and the other immediately upstream of the gene encoding the TCF4 transcription factor (Fig. S2, *B* and *D*).

**Figure 3:**
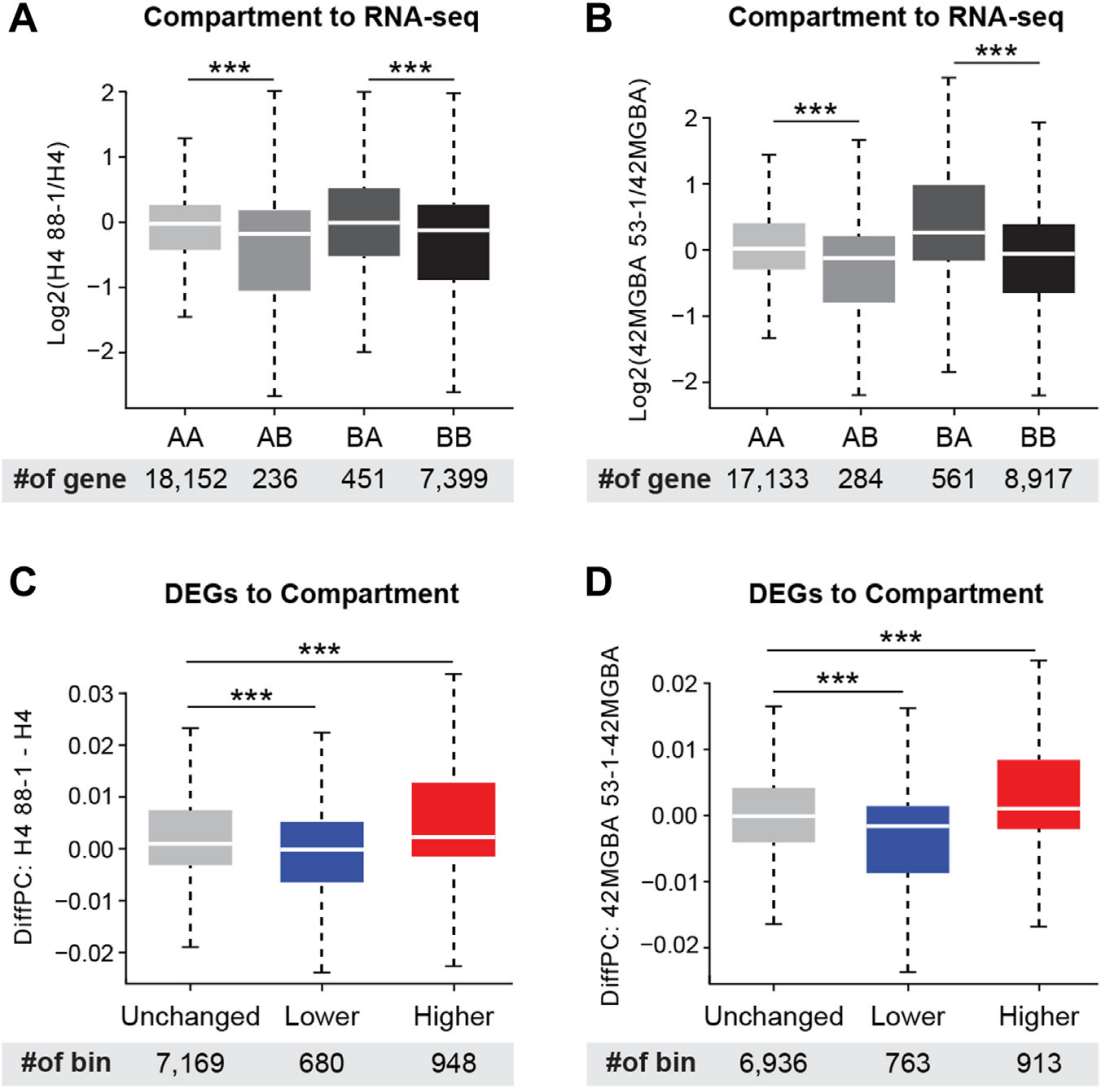
**Impact of compartment switching on gene expression.***A* and *B*, the effect of A/B compartment switching on the expression of genes within the switched compartments in the H4 (*A*) and 42MGBA (*B*) cell systems. *C* and *D*, enrichment of STAG2-regulated DEGs in switched compartments in the H4 (*C*) and 42MGBA (*D*) isogenic systems. ∗∗∗ indicates *p* < 0.001 using the Wilcoxon test.

Lastly, we integrated the compartment analysis with RNA-seq data from the same samples to determine the effect of compartment switching on gene expression. In both H4 and 42MGBA cells, the expression of genes in compartments switched from A to B was reduced after STAG2 correction whereas the expression of genes in compartments switched from B to A was increased (Fig. 3, *A* and *B*). These findings are consistent with the fact that A compartments are comprised of transcriptionally active euchromatin and B compartments are comprised of transcriptionally inactive heterochromatin. Conversely, genes whose expression was upregulated after STAG2 correction tended to be in compartments switched from B to A whereas genes whose expression was downregulated after STAG2 correction tended to be in compartments switched from A to B (Fig. 3, *C* and *D*).

### STAG2 is dispensable for the maintenance of TADs in human cells

Topologically associating domains (TADs) are regions of contiguous chromatin of ∼100 kb–2 Mb that interact with themselves more frequently than with other regions of chromatin on the same chromosome. Inactivation of cohesin subunits RAD21 and NIPBL has been shown to cause the immediate complete collapse of TADs (9, 10, 11, 12).To determine if STAG2 was involved in the maintenance of the TAD structure in human GBM cells, we performed TAD-level analyses of the Hi-C data. Stable correction of STAG2 mutations in H4 and in 42MGBA cells had no effect on TAD structure (Fig. S3, *A–D*) and there was no consistent difference between TAD structures in GBM PDXs with wild-type STAG2 and those with mutant STAG2 (Fig. S3*E*).

### Identification of STAG2-regulated dynamic chromatin loops in GBM cells

**Figure 4:**
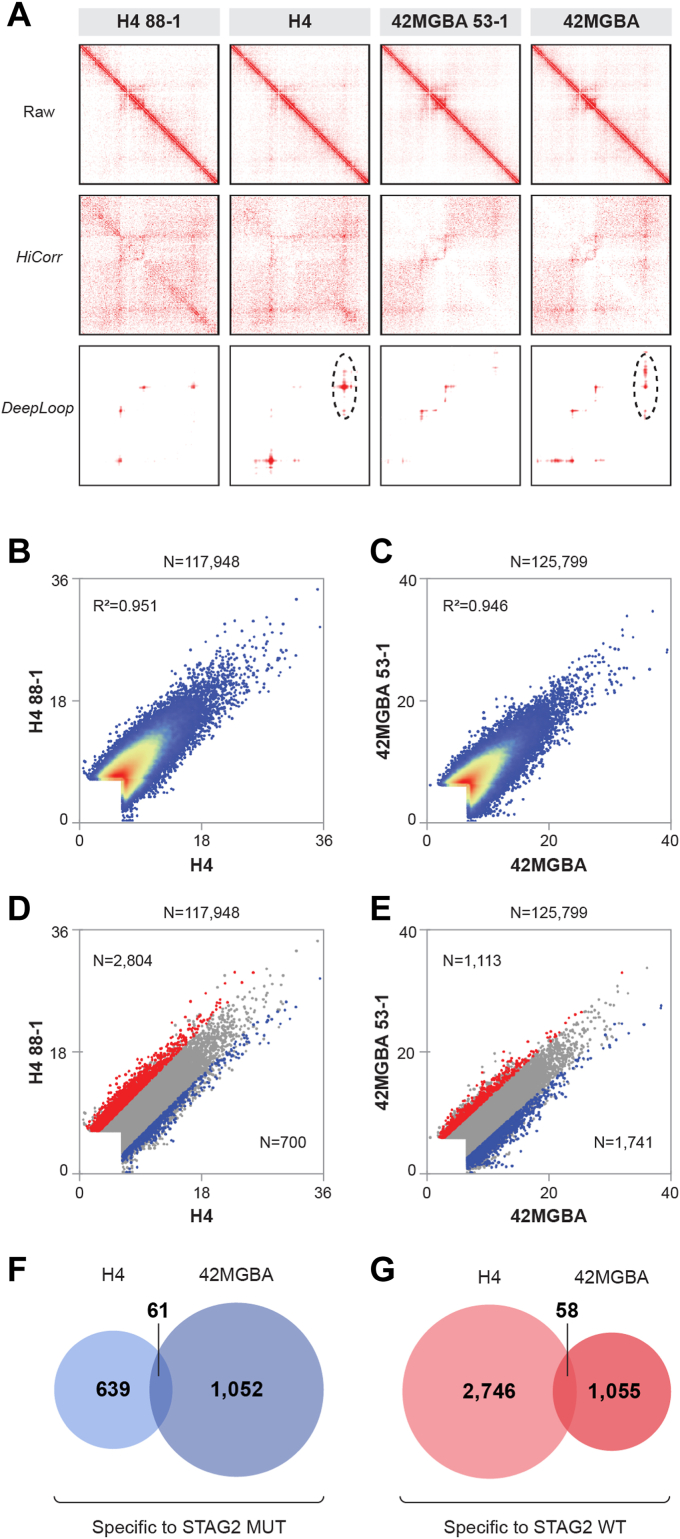
**Impact of STAG2 mutations on chromatin loops in GBM cells.***A*, Hi-C heatmaps showing the effects of the *HiCorr* and *DeepLoop* packages on chromatin loop identification (5 kb resolution). The *top row* shows raw contact matrices for a representative 1.1 Mb region on chromosome 21 for the H4 and 42MGBA isogenic cells. The *middle row* shows the effect of bias correction using HiCorr, which reveals subTAD chromatin interactions with high sensitivity. The *bottom row* shows the effect of *DeepLoop* on removing noise and enhancing signal in *HiCorr* corrected heatmaps, leading to more robust and specific chromatin loop calls. An example of a chromatin loop specific to STAG2-mutant cells is circled. *B* and *C*, scatterplots showing the effect of STAG2 on the intensity of the strongest ∼100,000 chromatin loops in the (*B*) H4 and (*C*) 42MGBA isogenic systems, demonstrating a high level of correlation between STAG2-mutant and wild-type isogenic cells. Total number of loops and R-squared are shown. *D* and *E*, same as (*B*) and (*C*) except that *blue pixels* represent loops specific to STAG2-mutant cells and *red pixels* represent loops that are specific to STAG2 wild-type cells (*p* < 0.05). *F* and *G*, Venn diagrams showing the numbers of chromatin loops that are specific to STAG2-mutant and STAG2 wild-type cells in the two experimental systems as well as the numbers of overlaps between the two experimental systems.

We next investigated whether correcting STAG2 mutations would alter individual chromatin loops in GBM cells. To explore this, we analyzed the Hi-C data using our recently-developed *HiCorr* and *DeepLoop* pipelines, which greatly improve the sensitivity and quantitative nature of chromatin loop identification at kb resolution (25, 26). An example of the utility of *HiCorr* and *DeepLoop* for enhancing the sensitivity of chromatin loop identification in H4 and 42MGBA cells is shown in Figure 4*A*. After applying *HiCorr* and *DeepLoop* to our Hi-C datasets obtained from H4 and 42MGBA isogenic pairs of cells (Fig. 4*A*), we used a regression analysis to compare individual chromatin loops in the isogenic cells. We found that correction of mutant STAG2 did not cause massive, genome-wide gain or loss of all chromatin loops (Fig. 4, *B* and *C*). Instead, STAG2 correction altered a small minority of individual chromatin loops (shown in red and blue in Fig. 4, *E* and *D*).

There were 700 loops specific to H4 cells and 2804 loops specific to H4 88-1 cells (Fig. 4*F*). Similarly, there were 1113 loops specific to 42MGBA cells and 1113 loops specific to 42MGBA 53-1 cells (Fig. 4*G*). There was a clear difference in STAG2-regulated dynamic chromatin loops between H4 and 42MGBA cells. However, there were 61 shared mutant STAG2-specific loops and 58 shared wild-type STAG2-specific loops between these two cell systems (Fig. 4, *F* and *G*), which was 9.0-fold and 3.3-fold higher, respectively, than would be expected by chance alone (*p* < 0.01, hypergeometric test).

**Figure 5:**
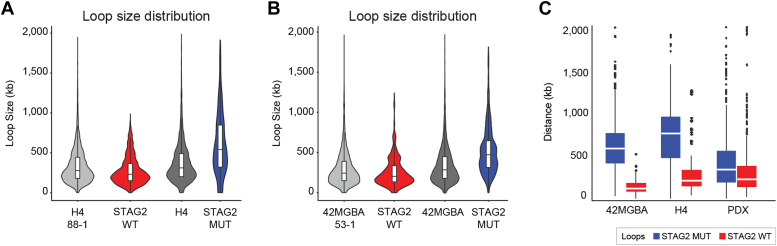
**Effect of STAG2 mutations on chromatin loop size in GBM cells and tumors.***A* and *B*, the distribution of chromatin loop sizes in the H4 and 42MGBA cell systems are shown. H4, H4 88-1, 42MGBA, and 42MGBA 53-1 refer to the loop size distributions for all loops identified in these cell lines, whereas “STAG2 WT” and “STAG2 MUT” refer to the loop size distributions for chromatin loops specific to STAG2 wild-type or mutant cells. *C*, box plots showing loop size distributions for the H4 and 42MGBA isogenic cells and the four GBM PDX tumors. *Red* represents loops specific to STAG2-mutant cells/tumors, and *blue* represents loops specific to STAG2 wild-type cells/tumors (fold change >4).

Next, we determined the effect of STAG2 correction on the *size* of chromatin loops in H4 and 42MGBA cells. When considering all loops, chromatin loop size was indistinguishable between parental and corrected cells (Fig. 5, *A* and *B*). However, there was a striking size difference when considering only STAG2-regulated chromatin loops—loops specific to STAG2-mutant cells were much larger (median size ∼700kb) than all loops and loops specific to the STAG2 wild-type cells (median size ∼200 kb; Fig. 5, *A* and *B*).

We next tested whether this STAG2 effect on chromatin loop size in cultured GBM cells was generalizable to genetically unmodified human GBM tumors. To do this we compared the size of chromatin loops enriched in STAG2-mutant GBM PDXs (PDX14, PDX44) to the size of chromatin loops enriched in STAG2 wild-type GBM PDXs (PDX6, PDX12). We found that loops enriched in STAG2-mutant tumors were significantly larger than loops enriched in STAG2 wild-type tumors (Fig. 5*C*), confirming the results from the isogenic cell culture systems.

### Identification of STAG2-regulated loop-gene combinations in GBM cells

**Figure 6:**
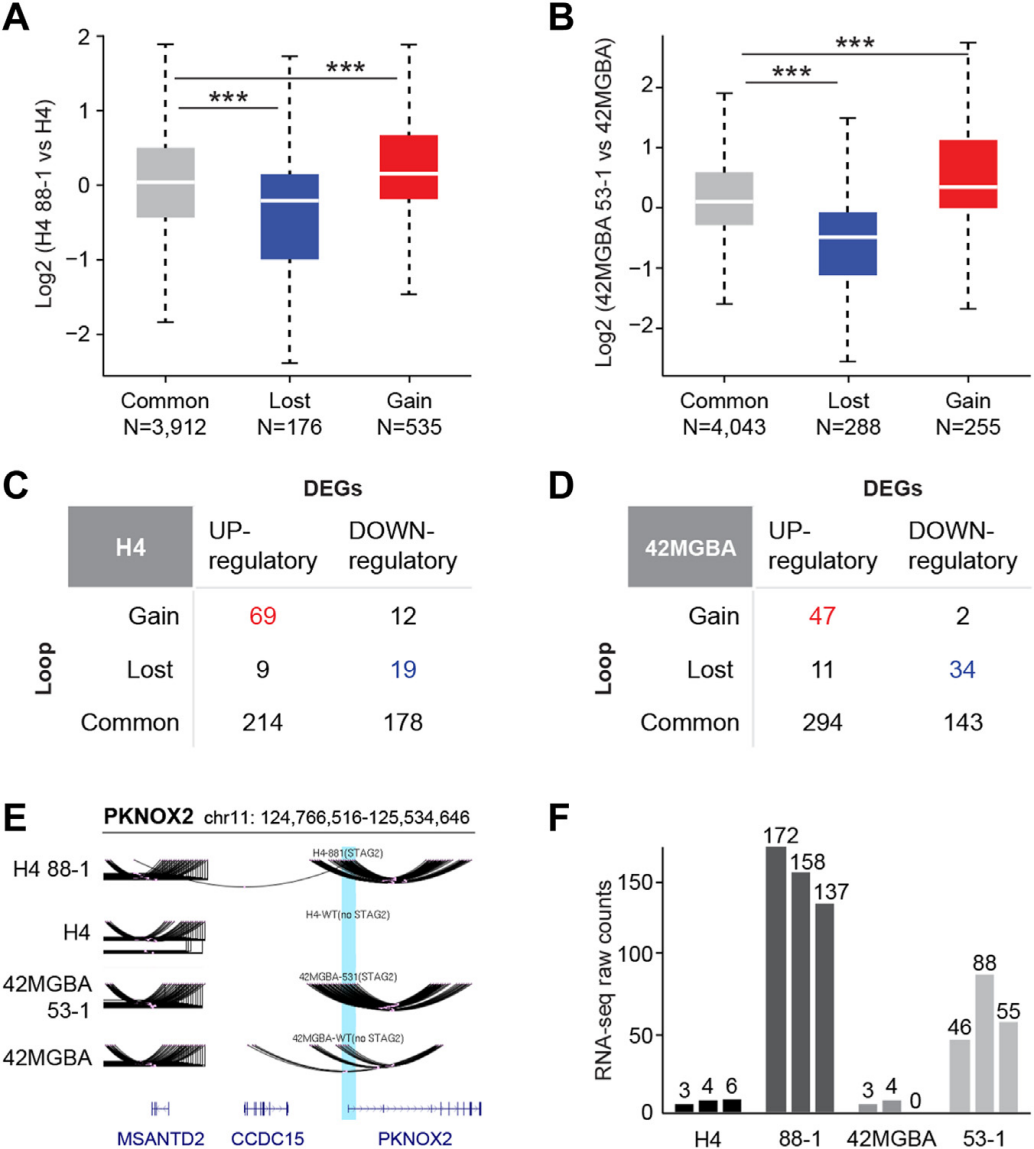
**Effect of STAG2-regulated chromatin loops on gene expression.***A* and *B*, boxplots showing the effect of STAG2 correction on the expression of genes adjacent to STAG2-regulated chromatin loops in (*A*) H4 and (*B*) 42MGBA systems (*p* < 0.001, Wilcoxon test). The numbers of loop/gene combinations is shown. “Lost” refers to loops that are lost after STAG2-correction, “Gain” refers to loops that were gained after STAG2-correction, and “Common” refers to loops for which STAG2 correction had no effect. *C* and *D*, tables showing the relationship of STAG2-regulated genes to nearby STAG2-regulated chromatin loops in the H4 and 42MGBA systems. The loop-gene combinations in *red* and *blue* are listed in Tables S8 and S9. *E*, depiction of the STAG2-regulated dynamic chromatin loop connecting the promoter of PKNOX2 with a downstream enhancer. *Blue bar* designates the transcriptional start site of PKNOX2. *F*, PKNOX2 gene expression in H4 and 42MGBA isogenic cells as measured by RNA-seq. Numbers of transcripts in three biological replicates of each cell line are shown.

Next, we integrated the Hi-C data with the RNA-seq data to determine, on a global scale, whether STAG2-regulated dynamic chromatin loops regulate the expression of adjacent genes (defined as having a loop anchor within 3 kb of the transcriptional start site). We found that chromatin loops that were lost after STAG2 correction tended to be accompanied by reduced expression of adjacent genes whereas loops that were gained after STAG2 correction tended to be accompanied by increased expression of adjacent genes (Fig. 6, *A* and *B*). This finding supports the model that STAG2-dependent chromatin loops activate the expression of adjacent genes that they regulate.

We then identified all individual STAG2-regulated genes that were accompanied by STAG2-regulated dynamic chromatin loops (Fig. 6, *C* and *D*). This analysis revealed that genes whose expression was upregulated by STAG2 correction tended to be accompanied by the gain of an adjacent chromatin loop whereas genes whose expression was downregulated by STAG2 correction tended to be accompanied by the loss of an adjacent chromatin loop (*p* < 0.05 for all comparisons, Fisher’s exact test). One prominent example is the PKNOX2 homeobox gene, which has a robust STAG2-regulated chromatin loop connecting its promoter to a previously unrecognized downstream enhancer and is robustly regulated by STAG2 in both isogenic systems (Fig. 6, *E* and *F*). For a list of all STAG2-regulated gene/loop combinations, see Tables S8 and S9. In addition to PKNOX2, two additional STAG2-regulated gene/loop combinations were conserved in both the H4 and 42MGBA cell systems—the endothelin-1 gene (EDN1) and the SH3RF3 gene—both have extensive reported connections to cancer (27, 28).

### STAG2 mutation activates polycomb signaling in GBM

**Figure 7:**
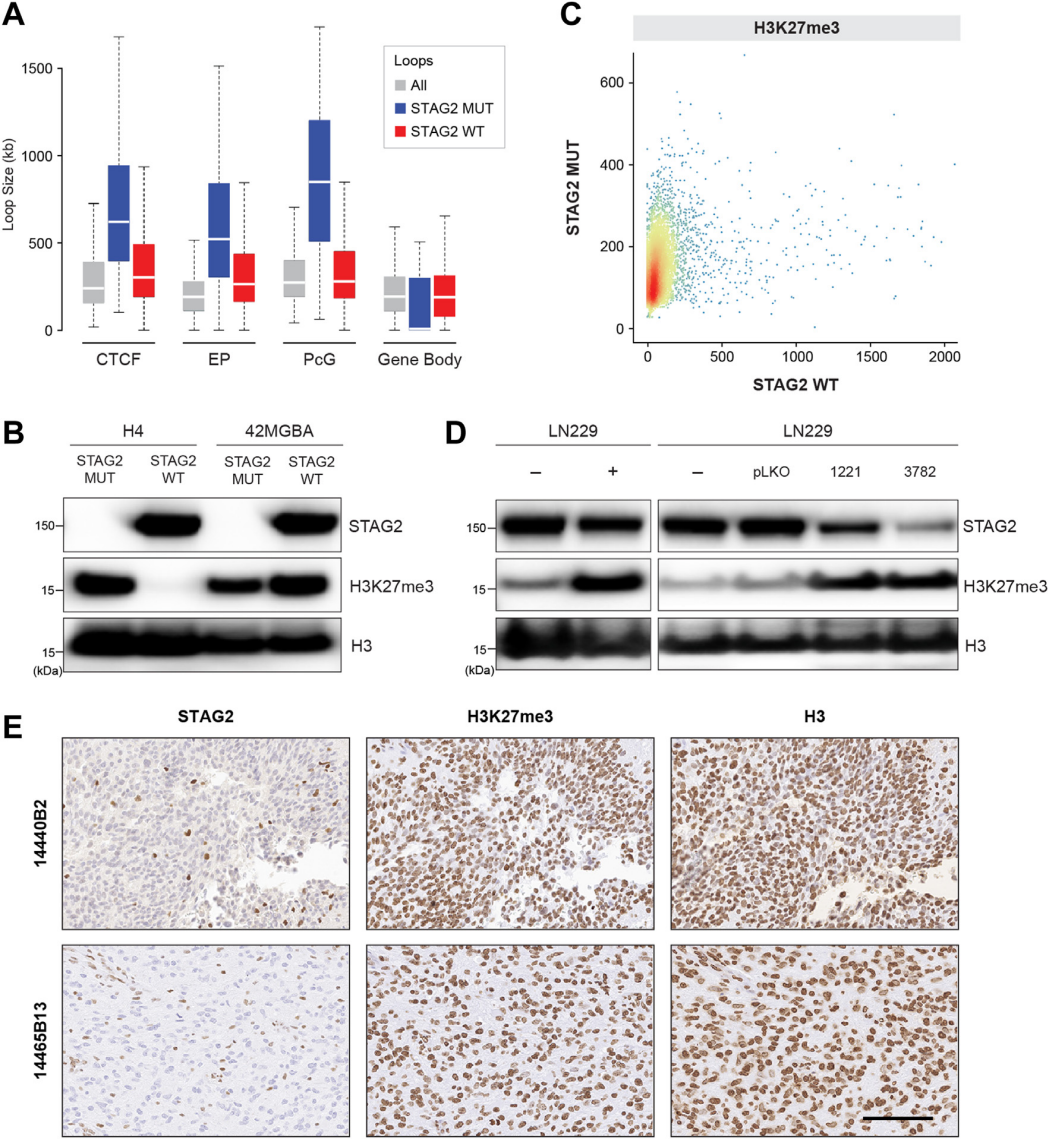
**Activation of Polycomb signaling in STAG2-mutant GBM cells and tumors.***A*, Hi-C data from H4 cells are integrated with publicly available ChIP-seq data from human astrocytes. *Gray boxes*–all loops; *Blue boxes*–loops specific to STAG2-mutant cells; *Red boxes*–loops specific to STAG2 wild-type cells. CTCF–CTCF; EP - Enhancer-Promoter (H3K4me3+H3K27Ac); PcG - Polycomb (H3K27me3); and Gene Body (H3K36me3). *B*, Western blot performed on chromatin extracts from H4, H4 88-1, 42MGBA, and 42MGBA 53-1 cells using the indicated antibodies. This experiment was performed twice. *C*, ChIP-seq performed on chromatin purified from H4 cells and H4 88-1 cells with H3K27me3 antibodies using a spike in control. The scatter plot shown depicts the intensities of individual H3K27me3 peaks in H4 cells and STAG2-corrected derivatives in Reads Per Kilobase/Million (RPKM). *D*, Western blots with the indicated antibodies performed on chromatin extracts from pooled clones of LN229 GBM cells (*left*) infected with (−) the empty lentiCRISPRv2 vector or (+) with the vector expressing a STAG2 gRNA and selected for 25 h in puromycin (1.0 ug/ml) and (*right*) infected with the pLKO shRNA lentiviral vector or the vector expressing one of two different STAG2 shRNAs and selected briefly in puromycin. This experiment was performed twice. *E*, immunohistochemistry with the antibodies indicated two different FFPE STAG2-mutant GBM primary tumors. Additional stained tumors are shown in Fig. S4. Scale bar is 100 um. This experiment was performed once.

Finally, we integrated our Hi-C data with publically available ChIP-seq from human astrocytes (the precursor cell type for GBM; GEO accession number GSE29611; ref (29)) to determine whether the enlarged loops specific to STAG2-mutant cells (Fig. 5*A*) contained CTCF binding sites or epigenetic marks. This analysis revealed that binding sites for the H3K27me Polycomb (PcG) mark were particularly enriched in the longest STAG2-specific loops (Fig. 7*A*), suggesting a connection between Polycomb signaling and STAG2-regulated dynamic chromatin loops. These data, when taken together with published studies indicating that cohesin can negatively regulate PcG signaling in model organisms (17, 18, 19, 20), suggested the hypothesis that STAG2 mutation could activate PcG signaling in GBM cells.

To test this hypothesis, Western blotting with antibodies to the H3K27me3 chromatin mark was performed on chromatin extracts from the H4 and 42MGBA isogenic systems. Remarkably, the H3K27me3 mark was completely extinguished by stable correction of mutant STAG2 in H4 cells (Fig. 7*B*), indicating that mutational inactivation of STAG2 can result in activation of PcG signaling in GBM. In contrast, there was no effect of STAG2 correction on PcG signaling in 42MGBA cells. To confirm and extend the finding in H4 cells, we next performed H3K27me3 ChIP-seq on H4 cells and H4 88-1 cells and found a robust H3K27me3 signal in H4 cells but no such signal in H4 88-1 cells (Fig. 7*C*), confirming the disappearance of the H3K27me3 chromatin mark after STAG2 correction in these cells.

Since there was a dramatic effect of STAG2 correction on PcG signaling in H4 cells but not in 42MGBA cells, we felt it was important to demonstrate the potential generality of this effect by identifying a second GBM cell line in which STAG2 inactivation resulted in activated PcG signaling. To do this, we generated an additional isogenic set of STAG2-proficient and deficient GBM cells by infecting LN229 GBM cells carrying the wild-type STAG2 with either a high-efficiency STAG2 CRISPR KO lentivirus or vector alone. We found that acute inactivation of STAG2 in LN229 cells substantially increased the H3K27me3 chromatin mark (Fig. 7*D*), confirming the results in H4 cells in a second cell line. Similar results were obtained after STAG2 knockdown in LN229 cells by infecting lentiviruses expressing STAG2 shRNAs (Fig. 7*D*).

We next investigated whether we could detect high levels of the H3K27me3 chromatin mark in genetically unmodified primary GBM tumors with STAG2 mutations. To examine this, we performed immunohistochemistry using H3K27me3, STAG2, and total H3 antibodies on four primary GBM tumors with STAG2 mutations (30). Each of the STAG2-mutant GBM tumors (which fail to express STAG2 protein) expressed high levels of nuclear H3K27me3 (Figs. 7*E* and S4), providing further support for the connection between STAG2 mutations and PcG signaling in GBM.

**Figure 8:**
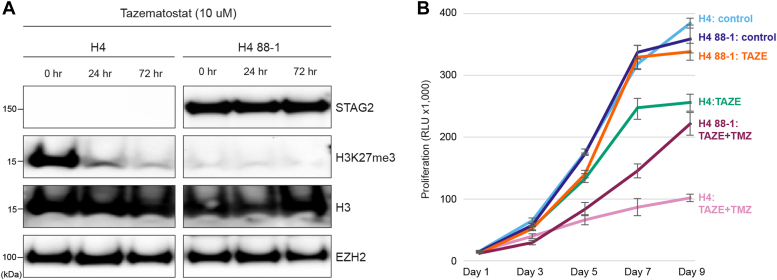
**STAG2 can modulate sensitivity to pharmacologic EZH2 inhibition in GBM.***A*, Western blot performed on chromatin extracts prepared from H4 cells and H4 88-1 STAG2-corrected derivatives treated with the EZH2 inhibitor Tazemetostat (10 uM) for the indicated time periods using the antibodies indicated. This experiment was performed twice. *B*, cellular proliferation of H4 cells and H4 88-1 STAG2-corrected derivatives treated with Tazemetostat (TAZE; 10 uM) and the alkylating agent temozolomide (TMZ; 200 uM) for the indicated time periods measured using the Cell Titer Glo assay. Error bars represent the standard deviation from triplicate wells.

Because inactivation of STAG2 appeared to result in activation of PcG signaling in GBM cells and tumors, we wondered whether STAG2-mutant cells would be more sensitive to PcG inhibitors. To test this hypothesis, we performed Western blot and *in vitro* proliferation assays on H4 cells and H4 88-1 cells treated with the clinical EZH2 inhibitor tazemetostat, which is FDA-approved for the treatment of lymphoma and sarcoma. EZH2 is the PcG PRC2 subunit that generates the H3K27me3 chromatin mark (31). Treatment with tazemetostat phenocopied STAG2 correction in H4 cells that it completely extinguished the H3K27me3 chromatin mark (Fig. 8*A*). To determine if this finding uncovered a potentially therapeutic vulnerability of STAG2 mutant GBM cells, we measured the effect of tazemetostat on the proliferation of H4 and H4 88-1 cells. We found that tazemetostat preferentially inhibited the proliferation of H4 cells, both as a single agent and when administered together with temozolomide, a standard-of-care therapy for GBM (Fig. 8*B*).

## Discussion

Here we report the effect of stable correction of naturally occurring, tumor-derived mutations of STAG2 on global gene expression, 3D genome organization, and Polycomb signaling in human GBM cells. We find that STAG2 mutation correction (i) alters the expression of about 10% of all expressed genes; (ii) alters a fraction of A/B compartment assignments and the expression of the genes within them, but does not affect TADs; (iii) alters the size and strength of individual chromatin loops throughout the genome, a subset of which regulate the expression of adjacent genes; and (iv) modulates Polycomb signaling in some human GBM cells.

The breadth and magnitude of the effect of modulating cohesin on gene expression is controversial (2). Using RNA-seq we demonstrate that stable correction of STAG2 mutations has a widespread impact on gene expression in human cancer cells. We find that the expression of about 10% of all genes is significantly altered by the correction of mutant STAG2 in each of the two GBM experimental systems studied. Virtually all the genes whose expression changed *the most* are downregulated after STAG2 correction (*i.e.*, upregulated by mutational inactivation of STAG2). Many of these genes are appealing putative effectors of STAG2 tumor suppression. For example, FGF7 (upregulated 100-fold in STAG2-mutant H4 cells) is a growth factor well known to promote proliferation and invasion (32), c-KIT (upregulated 300-fold in STAG2-mutant H4 cells) is a growth factor receptor, stem cell marker, and known oncogene (33), and MAGEC2 and MAGEA12 (upregulated >1000-fold and >300-fold in STAG2-mutant H4 cells, respectively) are “cancer/testis antigens” that are normally expressed specifically in male germ cells but are frequently dramatically upregulated in cancer cells *via* unknown mechanisms (34). While most of these genes were not previously known to be regulated by STAG2 or cohesin, Antony *et al.* previously reported that STAG2 regulates c-KIT expression in leukemia (35).

We also report the use of Hi-C to evaluate the effect of STAG2 correction on large-scale features of 3D genome organization in human cancer cells. We find no effect of stable STAG2 correction on TAD structure in GBM, a finding consistent with previous results obtained in both neoplastic and non-neoplastic model systems, including in other cancer types that frequently harbor STAG2 mutations (36, 37, 38). However, we do find that ∼5% of A/B compartments are switched after correction of STAG2 mutations in human GBM cells, a finding consistent with several prior studies performed in other model systems (39, 40). Several of these compartment switches are conserved in both experiment systems studied – one adjacent to the corrected STAG2 gene itself and another adjacent to the TCF4 gene, leading to upregulation of TCF4 expression. The TCF4 gene is a known oncogene and encodes a key component of the Wnt signaling pathway (41), supporting a recent study pointing to mechanistic connections between the cohesin and Wnt signaling pathways in the pathogenesis of cancer (42).

Our Hi-C data also reveal that there is no *general* effect of STAG2 mutation on the global chromatin loop landscape in human GBM cells. This finding is not unexpected, because if STAG2 inactivation *did* result in the global collapse of chromatin loops, cancer cells with STAG2 truncating mutations would lack chromatin loops, which would almost certainly be incompatible with cellular viability. However, STAG2 correction does alter thousands of *individual* chromatin loops, a substantial number of which appear to regulate the expression of adjacent genes. PKNOX2 - a member of the Three Amino Acid Loop Extension (TALE) class of homeodomain proteins (43) - is one such newly discovered STAG2-regulated gene controlled by a STAG2-regulated chromatin loop. PKNOX2 is particularly promising as a putative STAG2 effector because of the known role of cohesin and STAG2 in controlling cellular differentiation.

Interestingly, the chromatin loops that are present specifically in STAG2-mutant cells are very large—significantly larger than loops in general and those are present specifically in STAG wild-type cells (35, 44). This finding is consistent with recent data indicating that STAG1-containing cohesin complexes (which predominate in STAG2-mutant cells; ref. (45)) have greater loop extrusion processivity than STAG2-containing cohesin complexes (15). This finding is also reminiscent of the known effect of inactivation of the WAPL subunit of cohesin on chromatin loop size (11). Importantly, for the first time, we also demonstrate this finding in primary human tumors, raising the possibility that large loops are a fundamental feature of STAG2-mutant cancers. Hi-C data from more primary tumor samples from a variety of cancer types are needed to confirm this intriguing possibility.

Our Hi-C data also led us to discover that mutation of STAG2 can lead to activation of PcG signaling in GBM, extending prior reports in model organisms to human cancer cells (16, 17, 18, 19, 20). This activation of PcG signaling led to enhanced sensitivity to EZH2 inhibitors, a finding that points to activated Polycomb signaling as a putative druggable effector of STAG2 tumor suppression. A previous study performed in Ewing sarcoma cells also pointed out a connection between cohesin mutation and PcG signaling in cancer cells; however, in that study, STAG2 mutation resulted in a diminution *of* PcG signaling, not the enhancement demonstrated here (46, 47). Since PcG is generally considered to be an oncogenic pathway (48), this prior finding was unexpected, and—when taken together with the work presented here—suggest that there may be tumor type-specific differences in the mechanisms of cohesin tumor suppression. Finally, as a potential cancer drug target, it would be interesting to determine whether the disruptive effect of STAG2 mutations on 3D genome organization and epigenomics can be overcome by PRC2 complex inhibitors.

An important strength of this work is that we study the effect of the endogenous, naturally occurring truncating alleles of STAG2 in a biologically and clinically relevant cancer type. Another strength is that we use recently developed algorithms *HiCorr* and *DeepLoop* to enhance the sensitivity of chromatin loop detection from Hi-C data. However, a weakness of this study is that since there is still no functional assay available for measuring STAG2 tumor suppression after reconstitution of wild-type STAG2 in cells with naturally occurring STAG2 mutations, it is not possible to determine the extent to which the STAG2-regulated genes and chromatin loops identified herein are required as effectors of STAG2 tumor suppression in human GBM cells.

In summary, here we provide a comprehensive census of STAG2-regulated genes and features of 3D genome organization in human GBM, identifying a host of novel putative effectors of STAG2 tumor suppression and potential targets for therapeutic intervention. Furthermore, by illuminating the landscape of STAG2-regulated genes, A/B compartments, chromatin loops, and pathways in GBM, these data provide important clues into the still largely mysterious mechanism of STAG2 tumor suppression in human cancer.

## Experimental procedures

### Cell lines

H4 cells and LN229 cells were obtained from ATCC. 42MGBA cells were obtained from the DSMZ-German Collection of Microorganisms and Cell Cultures. Gene-edited derivatives of H4 and 42MGBA cells were generated as described in ref. (3). All cell lines were maintained in Dulbecco's modified Eagle's medium supplemented with 10% fetal bovine serum and 1% Pen/Strep at 37 °C in 5% CO2.

### RNA-seq

Total RNA was isolated using the RNeasy Mini Kit (Qiagen). RNA-seq libraries were prepared using the NEB Next Ultra II RNA Library Prep Kit (NEB) and sequenced on an Illumina NovaSeq 6000 instrument. RNA-seq data were aligned to the human reference genome (hg19) by using HISAT2 (49), and read counts for each gene are called using featureCounts (50). The statistical tests for RNA-seq data, normalizing raw data and identifying differentially expressed genes in this study were derived from a statistical model derived from DESeq2 (51).

### qRT-PCR

Total RNA was prepared by standard TRIZOL-based methods. Quantitative reverse transcription-PCR (qRT-PCR) was performed in StepOnePlus Real- Foster City, CA and the Superscript III Platinum One Step qRT-PCR System (Invitrogen), according to the manufacturers' specifications. Relative gene expression levels were calculated using the 2^−Δ(ΔCT)^ method, normalizing the expression of the GAPDH housekeeping gene. All assays were performed at least in triplicate.

### Western blot

Protein lysates were dissolved in LDS sample buffer, boiled for 5 min, and separated by SDS-PAGE. Proteins were transferred to polyvinylidene difluoride membranes, which were then probed with a 1:1000 dilution of primary antibodies rotating overnight at 4 °C (STAG2—Santa Cruz 81852, HEPH—Santa Cruz 365365, H3K27me3—Cell Signaling 9733, H3 - Cell Signaling 14269, EZH2—Cell Signaling 5246, and GAPDH—Cell Signaling 2118). After incubation with horseradish peroxidase-conjugated secondary antibodies (Cell Signaling) rotating for 1 h at room temperature, membranes were developed with SuperSignal West Pico PLUS chemiluminescent substrate (Pierce) and imaged using a myECL imager (Pierce).

### Hi-C

Hi-C libraries were generated using the Arima Hi-C+ kit (Arima Genomics) as described in the kit instructions. Libraries were sequenced on an Illumina NovaSeq 6000 instrument by paired-end sequencing. Full-length paired-end reads were trimmed to 36 bp and then aligned to the reference human genome (hg19) using bowtie in parallel (52). Next, two SAM files for each end were merged into a single file, and only uniquely mapped pair-end reads (∼60% of the total) were retained. Next, read pairs that shared the same chromosome and start sites were removed. After removing PCR duplications, we assigned read pairs to GANTC-digested fragments and discarded read pairs located within the same fragment. Next, based on strand information, we assigned the remaining reads to three categories - inward, outward and same-strand. We kept “inward” read pairs if the distance between two fragments was >1kb, and “outward” reads pairs is the distance was >5kb. Then, we merged filtered “inward”, “outward” and all “same-strand” together as cis read pairs. After filtering steps, we obtained cis and trans fragment pairs. Since the analysis pipeline we typically use is at 5kb resolution, we then assign each fragment pair to 5kb anchor pairs. We then applied the *HiCorr* bias correction algorithm to the data as described in ref. (25) followed by the LoopEnhance tool from *DeepLoop* to enhance low depth data and remove noise pixels (26). For all pair-wise comparisons from STAG2 wild-type and STAG2-mutant samples, we merged the top 100k loops from each condition, generating ∼120k loops in total. We then compared the strength of these loops in STAG2-mutant and STAG2-corrected samples. Using a simple Z-score cutoff with *p*-value <0.05 (two-side), we identified lost, gained and common loops.

### ChIP-seq

Crosslinked chromatin was prepared using the SimpleChIP Plus Sonication Chromatin IP Kit (Cell Signaling) and ChIP-seq libraries were prepared using the DNA Library Prep Kit for Illumina (Cell Signaling). Libraries were sequenced on an Illumina NovaSeq 6000 instrument. For quantitative comparisons of ChIP-seq data from different samples, the spike in control normalization (53) was performed using *Drosophila* Spike-In Chromatin and the Spike-In Antibody, both from ActiveMotif. To do this, Spike-in ChIP-seq data was mapped to a human reference genome (hg19) and Spike-in ChIP-seq data was mapped to a *Drosophila* reference genome (dm6). We then counted uniquely aligning *Drosophila* sequence tags and identified the sample containing the least number of tags. We then compared *Drosophila* tag counts from other samples to the sample containing the least tags and generated a normalization factor for each comparison. (Sample 1 with lowest tag count/Sample 2) = Normalization factor.

### Lentiviral CRISPR and shRNA

To generate a high-efficiency STAG2 KO CRISPR lentivirus, we cloned ten different gRNAs targeting early exons of STAG2 into lentiCRISPRv2 (a gift from Feng Zhang, ref. (54)) and identified one that was able to introduce frameshift mutations into all alleles of STAG2 (>3) simultaneously in aneuploid human GBM cells with particularly high efficiency (>50%). This virus was then used to infect LN229 cells, infected cells were selected briefly in puromycin (1.0 ug/ml; 48 h), and cells were harvested in RIPA buffer for Western blot analysis. The generation and validation of high-efficiency STAG2 lentiviral shRNAs was performed as described in ref. (3).

### Immunohistochemistry

Immunohistochemistry was performed in the Georgetown University Medical Center Histopathology and Tissue Shared Resource. Five-micron sections from formalin-fixed paraffin-embedded tissues were de-paraffinized with xylene and rehydrated through a graded alcohol series. Heat-induced epitope retrieval (HIER) was performed by immersing the tissue sections at 98 °C for 20 min in a Target retrieval solution, high pH (Dako). Immunohistochemical staining was performed using the VectaStain Kit from Vector Labs according to the manufacturer’s instructions. Briefly, slides were treated with 3% hydrogen peroxide for 10 min. Endogenous biotin was blocked using an avidin/biotin blocking kit from Invitrogen. The slides were then treated with 10% normal goat serum for 10 min and exposed to primary antibodies for STAG2 (1:50, Santa Cruz, sc81852), H3 (1:500, Cell Signaling, #14269), or H3K27me3 (1:200, Cell Signaling, #9733) for 1 h at room temperature. Slides were then exposed to biotin-conjugated mouse secondary antibody (Vector Labs), Vectastain ABC reagent, and DAB chromagen (Dako). Slides were counterstained with hematoxylin (Fisher, Harris Modified Hematoxylin) at a 1:8 dilution for 2 min at room temperature, blued in 1% ammonium hydroxide for 1 min at room temperature, dehydrated, and mounted with Acrymount.

## Data availability

All raw RNA-seq and H-C sequencing data have been submitted to the Gene Expression Omnibus (GEO) under accession number GSE240343. *HiCorr* and *DeepLoop* pipelines for chromatin loop identification is available at https://github.com/JinLabBioinfo.

## Supporting information

This article contains supporting information.

## Conflict of interest

The authors declare that they have no known competing financial interests or personal relationships that could have appeared to influence the work reported in this paper.

## Supporting information

Supplemental Figures S1−S4

Supplemental Tables S1−S9
