# Supplementary material for: STAG2 mutations regulate 3D genome organization, chromatin loops, and Polycomb signaling in glioblastoma multiforme
Source: J Biol Chem. 2024 May 3;300(6):107341. doi: 10.1016/j.jbc.2024.107341 (PMC11157269; doi:10.1016/j.jbc.2024.107341)
Supplement: Supplemental Figures S1−S4 [file mmc1.docx]

**
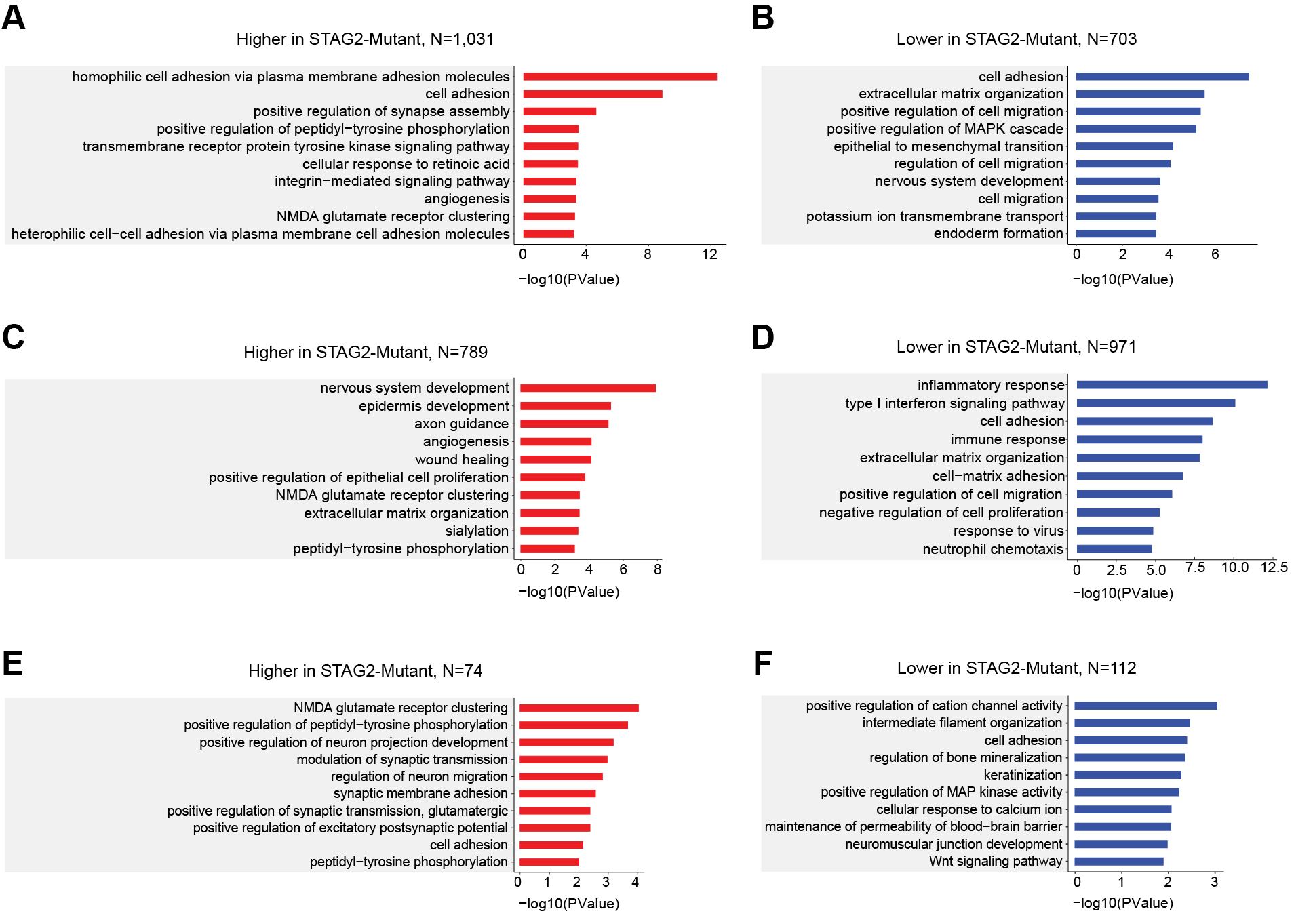
**

**Supplementary Figure S1.** Gene enrichment analysis of STAG2-regulated genes in (*A,B*) the H4 cell system, (*C,D*) the 42MGBA cell systems, and (*E*,*F*) the genes whose regulation by STAG2 was conserved between the H4 and 42MGBA cell systems.

**
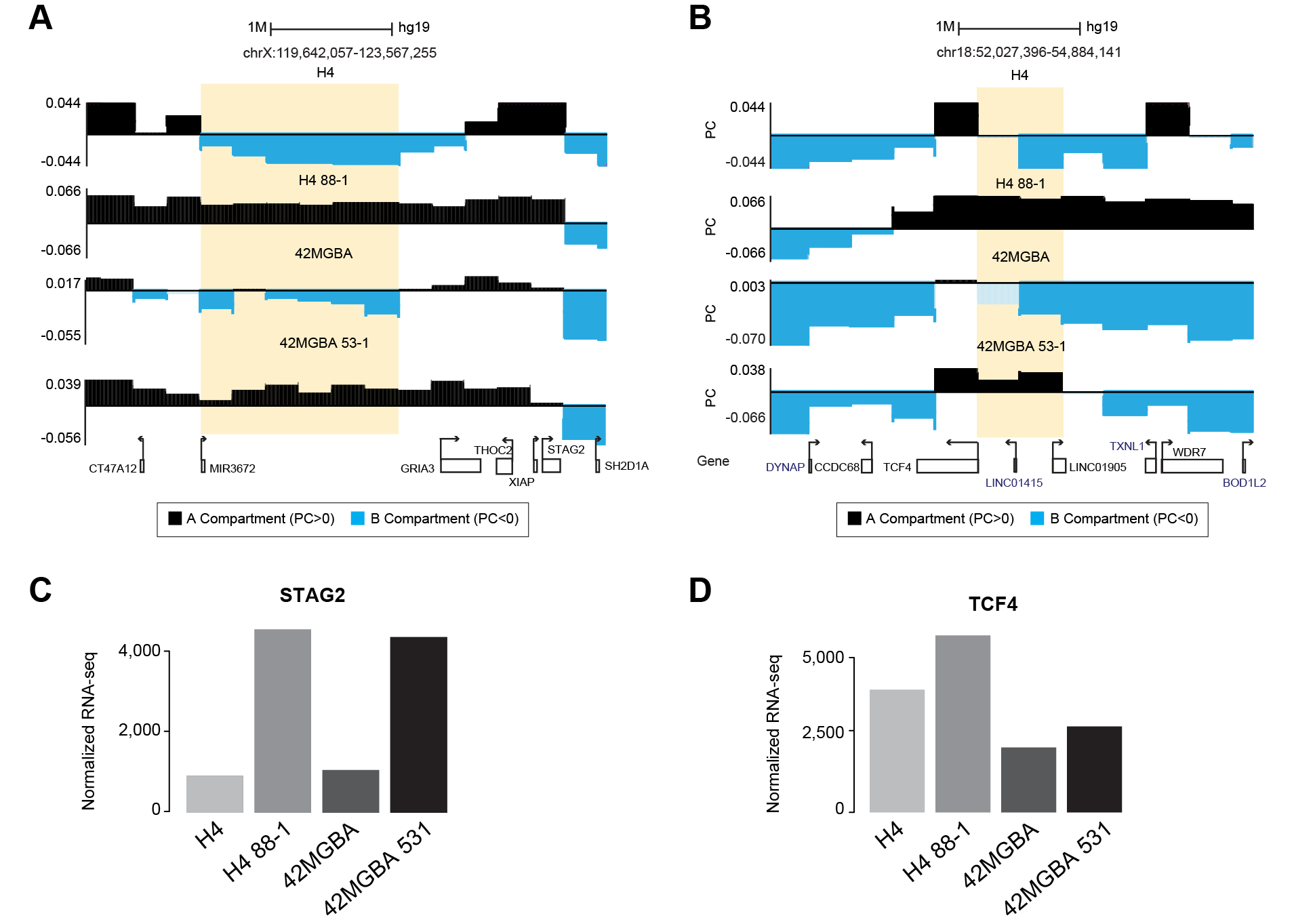
**

**Supplementary Figure S2.** Conserved STAG2-regulated compartments switches. (*A*,*B*) Compartment diagrams depicting the effect of STAG2 correction on B to A compartment switching in the H4 and 42MGBA isogenic systems for the genomic regions containing (*A*) STAG2 and (*B*) TCF4. (*C*,*D*) Bar graphs showing RNA-seq data for the TCF4 and STAG2 genes in the H4 and 42MGBA isogenic systems, demonstrating that the conserved B to A compartment switches after STAG2 correction lead to the expected increase in gene expression. Units are Reads Per Kilobase/Million reads (RPKM)

**
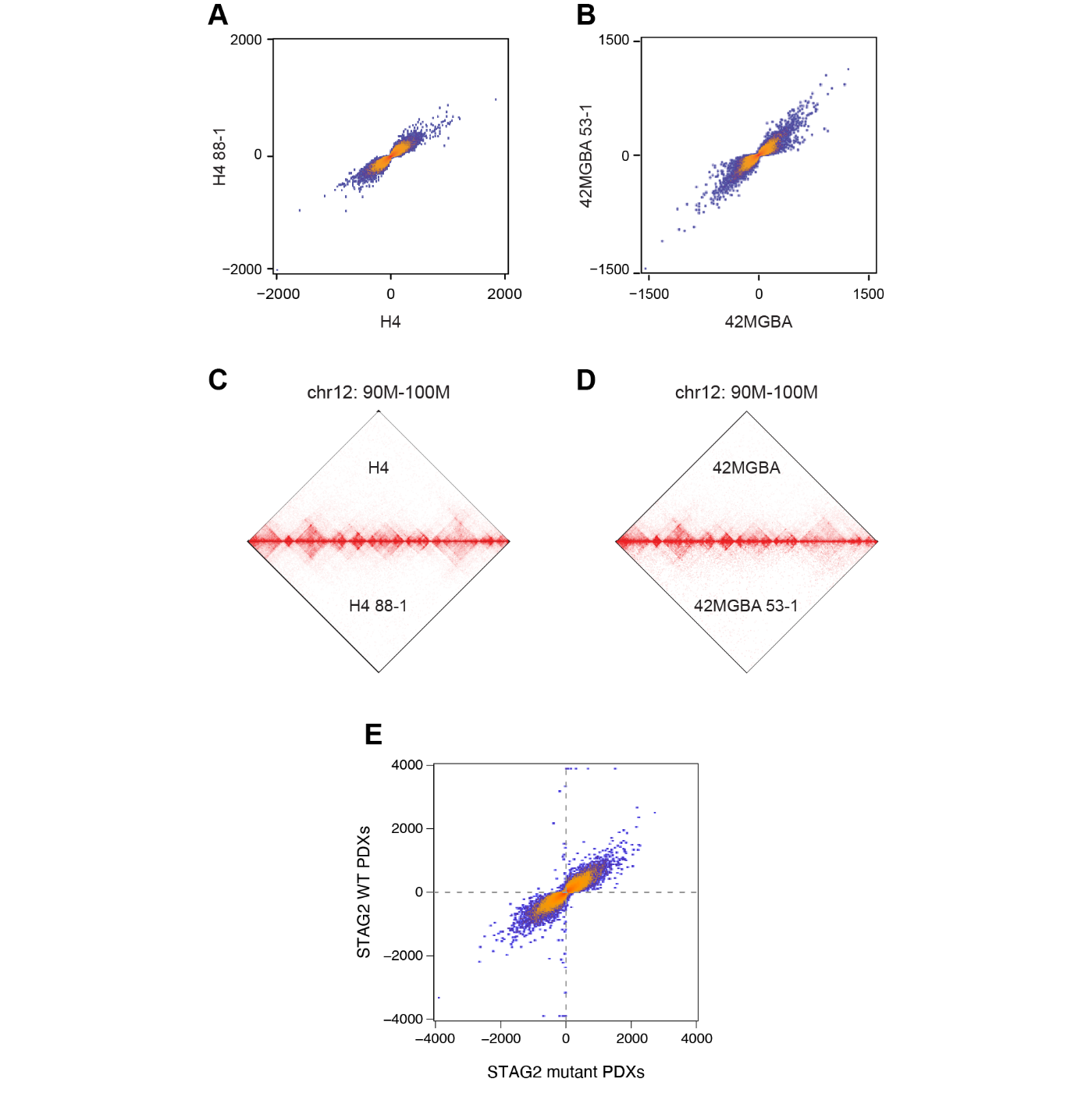
**

**Supplementary Figure S3**. Effect of STAG2 correction on TADs in GBM. (*A,B*) Scatterplots indicating the effect of STAG2 mutation correction on directionality indices (DI; a measurement of TAD boundaries) in the (*A*) H4 and (*B*) 42MGBA isogenic systems. (*C,D*) Examples of TAD heatmap contact matrices at 40 kb resolution for (*C*) H4 isogenic system and (*D*) 42MGBA isogenic systems for a representative 10 Mb genomic region on chromosome 12. (*E*) Scatterplots comparing TADs in STAG2 mutant GBM PDXs (x axis) with TADs in STAG2 wild-type GBM PDXs (y axis).


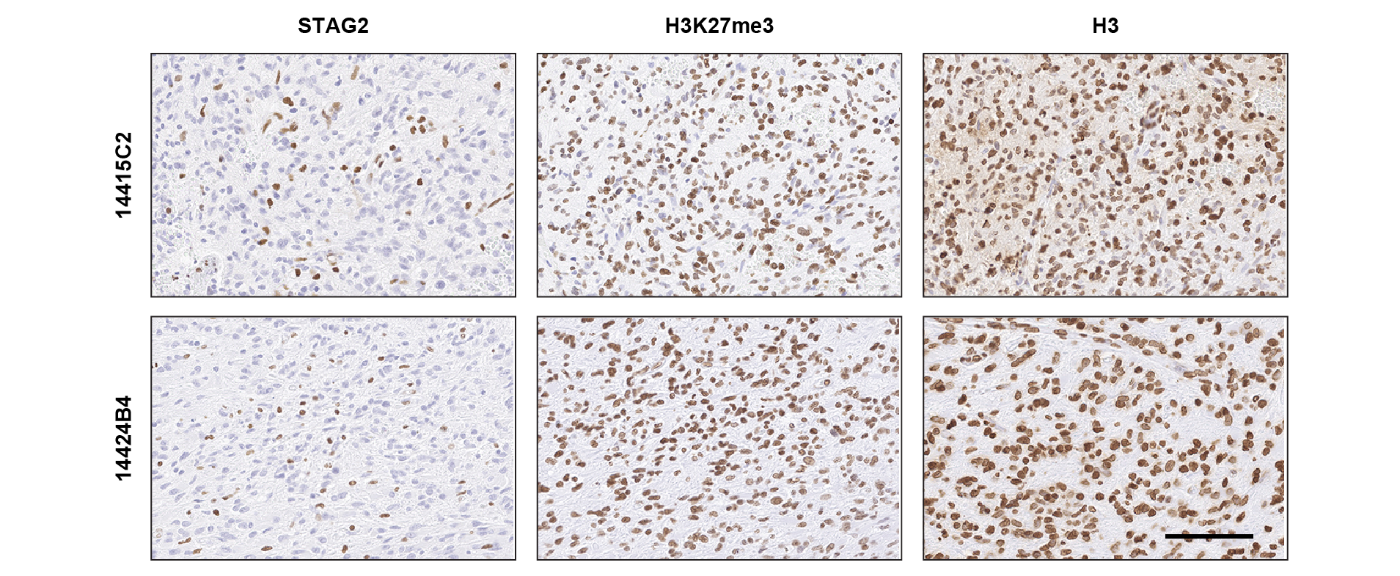


**Supplementary Figure S4**. Immunohistochemistry with the antibodies indicated to two additional FFPE STAG2-mutant GBM primary tumors. Scale bar is 100 μm. This experiment was performed once.
